# Potential Drug–Drug Interactions in Critically Ill Neonates: Association With Clinical Outcomes in a Prospective Cohort

**DOI:** 10.1155/ccrp/1033282

**Published:** 2026-10-14

**Authors:** Daniel Paiva Marques, Andreza Kelly Fernandes da Silva, Iris Ucella de Medeiros, Ramon Weyler Duarte Leopoldino, Antonio Gouveia Oliveira, Waldenice de Alencar Morais Lima, Rand Randall Martins

**Affiliations:** ^1^ Program in Pharmaceutical Services and Policies Health Science Center Federal University of Rio Grande do Norte Natal Brazil ufrn.br; ^2^ School Maternity Januário Cicco (MEJC/EBSERH) Health Science Center Federal University of Rio Grande do Norte Natal Brazil ufrn.br; ^3^ UFPI University Hospital (HU-UFPI/EBSERH) Health Science Center Federal University of Piaui Natal Brazil ufpi.br; ^4^ Program in Pharmaceutical Science Health Science Center Federal University of Rio Grande do Norte Natal Brazil ufrn.br

**Keywords:** drug interactions, drug-related side effects, neonatal intensive care units, newborn, prospective studies

## Abstract

**Background and Aims:**

Neonates admitted to neonatal intensive care units (NICUs) are frequently exposed to multiple medications, increasing the risk of potential drug–drug interactions (pDDIs). However, the clinical impact of these interactions remains poorly understood in this population. This study aimed to evaluate the occurrence of pDDIs in critically ill neonates and their association with clinical outcomes and laboratory parameters.

**Methods:**

This prospective cohort study included 365 neonates admitted to a NICU in Brazil between March 2023 and March 2024. Pharmacotherapy was monitored daily for the identification of pDDIs using the UpToDate database. Interactions were grouped according to their potential clinical outcomes and analyzed for their association with clinical and laboratory parameters. The evaluated parameters included heart rate, respiratory rate, blood pressure, creatinine clearance, urine output, and blood urea nitrogen.

**Results:**

A total of 255 neonates (69.9%) experienced at least one pDDI, most of which were classified as risk category C (88.0%). The most frequent mechanisms of pDDIs were associated with heart rate changes (27.4%), nephrotoxicity (18.3%), central nervous system (CNS) depression (14.8%), and hypotension (14.8%). Neonates with pDDIs associated with heart rate changes had higher heart rates (158.1 ± 16.7 vs. 148.7 ± 17.0 bpm; *p* < 0.001). Nephrotoxic pDDIs were associated with reduced creatinine clearance (14.4 ± 13.5 vs. 25.8 ± 18.2 mL/min/1.73 m^2^; *p* < 0.001) and higher blood urea nitrogen levels (52.9 ± 36.2 vs. 27.6 ± 24.4 mg/dL; *p* < 0.001). pDDIs associated with hypotension significantly reduced mean arterial pressure (51.1 ± 11.5 vs. 58.3 ± 11.8 mmHg; *p* < 0.001).

**Conclusion:**

pDDIs are frequent in NICUs and are associated with clinically relevant changes, particularly those involving an increased risk of higher heart rates, renal impairment, and hypotension.

## 1. Background

Approximately one in 10 live births worldwide occurs preterm [1], and preterm infants constitute an important proportion of neonates requiring specialized and intensive care. Neonates admitted to neonatal intensive care units (NICUs) frequently present clinical conditions that require complex pharmacotherapy. Drug treatment in this population is particularly challenging because of the rapid developmental changes in pharmacokinetics and pharmacodynamics that occur during the neonatal period [2], the widespread use of off‐label medications [3], and the increased vulnerability to adverse drug events, including drug–drug interactions (DDIs) [4].

DDIs occur when one drug modifies the pharmacokinetics or pharmacodynamics of another, including alterations in absorption, distribution, metabolism, or elimination, as well as additive or antagonistic pharmacodynamic effects, potentially affecting therapeutic efficacy or increasing toxicity [5]. Adult patients in intensive care units (ICUs) are especially susceptible, with approximately two‐thirds experiencing at least one potential drug–drug interactions (pDDI) [6]. In NICUs, the risk appears similarly high. A Brazilian NICU study reported that approximately 70% of neonates were exposed to at least one DDI during hospitalization, with 30% classified as major [7]. Comparable rates have been observed in NICUs in Spain (74%) [8] and India (66%) [9].

It is important to emphasize that not all identified DDIs lead to adverse events or clinically significant changes. Most published data in adult populations refer to pDDIs identified through prescription screening with drug interaction databases, without direct evaluation of clinical outcomes [5]. In neonates, evidence linking medication‐related interaction risks to measurable clinical outcomes remains limited. Previous initiatives, such as the Baby NINJA program, have demonstrated that systematic surveillance of concurrent nephrotoxic medication exposure can reduce nephrotoxic‐associated acute kidney injury (AKI) in NICU patients [10]. However, studies specifically evaluating pDDIs across different pharmacological mechanisms and relating them to corresponding clinical and laboratory parameters remain scarce. Additionally, a major limitation of the databases commonly used to classify DDIs is that most of the underlying literature does not specifically address the neonatal population [11].

Therefore, to address this critical gap in the literature, the present study was designed as a prospective cohort with daily pharmacotherapy monitoring to characterize DDIs in neonates and evaluate their association with clinical and laboratory parameters.

## 2. Patients and Methods

### 2.1. Study Design and Population

This prospective cohort study was conducted from March 2023 to March 2024 in the NICU of a referral maternity hospital for high‐risk pregnancies located in Natal, Brazil. The NICU comprises 20 beds and has an average of 500 admissions per year. All neonates hospitalized for at least 24 h were eligible. For neonates with more than one hospitalization during the study period, only the first hospitalization was included in the analysis, and subsequent readmissions were excluded to avoid duplicate inclusion of the same patient. Neonates admitted solely for diagnostic procedures were also excluded.

This study was approved by the Research Ethics Committee of Maternidade Escola Januário Cicco, in accordance with Resolution No. 466/12 of the Brazilian National Health Council (approval number 5867920) and conducted in accordance with the Declaration of Helsinki. Written informed consent was obtained from the legal guardians of all participants.

### 2.2. Data Collection and Study Variables

Neonates were characterized based on demographic and clinical variables, including sex, gestational age (weeks), birth weight (g), Apgar scores at 1 and 5 min, admission diagnosis, and length of hospital stay. Maternal data were also collected, including maternal age, parity, history of miscarriage, type of delivery, and pregnancy‐related comorbidities.

Throughout the entire hospitalization period, neonates were monitored daily for clinical parameters and for occurrence of DDIs, while laboratory parameters were recorded from routine clinical practice whenever available. The clinical parameters assessed included body temperature (°C), heart rate (bpm), respiratory rate (rpm), capillary blood glucose (mg/dL), number of daily bowel movements, urinary output (mL/kg/h), systolic and diastolic blood pressure (DBP), and mean arterial pressure (MAP). MAP was calculated using the standard formula:

(1) MAP=SBP+2×DBP3,

where SBP represents systolic blood pressure and DBP.

The laboratory parameters assessed included hemoglobin, hematocrit, platelet count, leukocyte count, total and fractionated bilirubin, C‐reactive protein (CRP), sodium, potassium, calcium, phosphorus, serum creatinine, urea, aspartate aminotransferase (AST), and alanine aminotransferase (ALT). For parameters measured more than once per day, the daily mean value was considered.

Renal function was estimated by calculating creatinine clearance using the modified Schwartz formula for neonates:

(2) ClearancemL×min−11.73m2=k×lenght cmserum creatininemg×dL−1,

…using a *k* value of 0.33 for neonates, as originally proposed for preterm infants [12]. Body length was obtained from electronic medical records or documented by the nursing staff.

In cases where clinical or laboratory data were missing for a given day, values were neither imputed nor carried forward from previous measurements. Such data were treated as missing and excluded from the corresponding statistical analysis for that specific day. This approach was adopted to preserve analytical accuracy and minimize measurement bias.

pDDIs were identified daily through prescription review using the UpToDate platform (Wolters Kluwer), focusing on the detection of interactions between drug pairs. Only DDIs classified as Category C (monitoring recommended), D (therapy modification suggested), or X (combination contraindicated) were considered. Identified interactions were grouped based on their mechanisms of action and their potential clinical and laboratory consequences. For pDDIs potentially associated with CNS depression, heart rate and respiratory rate were evaluated as measurable clinical parameters; CNS depression itself was not assessed as a specific clinical diagnosis or graded outcome.

The daily monitoring was carried out by a multidisciplinary team consisting of the principal investigator, a clinical pharmacist, and two trained undergraduate pharmacy students. A pilot study involving 10 patients was conducted prior to data collection to validate the data collection instruments and ensure methodological consistency across team members.

### 2.3. Statistical Analysis

Data were analyzed using Stata software, Version 15.0 (Stata Corporation, College Station, TX, USA). In the descriptive analysis, neonatal characteristics were presented as relative and absolute frequencies, means and standard deviations, or medians and interquartile ranges (25th–75th percentiles), depending on the type of variable.

For comparisons between neonates with and without pDDIs, continuous variables were analyzed using Student’s *t*‐test or the Mann–Whitney test, as appropriate, and categorical variables were compared using the chi‐square test or Fisher’s exact test. A *p* value < 0.05 was considered statistically significant.

DDIs with similar potential outcomes were grouped, and their incidence rates were reported along with corresponding 95% confidence intervals (95% CIs). To assess the association between length of hospitalization and the incidence rate of DDIs grouped by potential outcomes, a mixed‐effects linear regression model was applied. The incidence rate was used as the dependent variable, while length of hospital stay was included as a fixed‐effect independent variable. Intraindividual variability was modeled as the random‐effect component. *p* values < 0.05 were considered statistically significant.

To explore the association between DDIs and selected clinical and laboratory parameters, Student’s *t*‐test was used to compare the means of the following parameters: heart rate, respiratory rate, blood pressure, creatinine clearance, urine output, and blood urea nitrogen levels (*p* < 0.05).

## 3. Results

Between March 2023 and March 2024, a total of 533 patients were admitted to the NICU. After excluding 168 neonates who were hospitalized for less than 24 h, were readmitted during the study period, or did not receive any medications, the final study sample comprised 365 patients.

Population characteristics are summarized in Table 1. No significant differences were observed between neonates with and without pDDIs regarding maternal characteristics. However, neonates exposed to pDDIs had significantly lower gestational age and birth weight compared to those without pDDIs (33.1 ± 4.2 vs. 35.4 ± 2.4 weeks and 1988.7 ± 930.8 vs. 2545.1 ± 892.5 g, respectively; both *p* < 0.001). Additionally, neonates with pDDIs had a longer length of hospital stay (19.8 ± 15.1 vs. 7.4 ± 5.7 days; *p* < 0.001) and higher mortality rates (12.4% vs. 1.8%; *p* < 0.001).

**TABLE 1:** Characteristics of the study population.

**Variable**	**Values**				***p***
	**No DDI (*n* = 110)**		**With DDI (*n* = 255)**		
Maternal age, years (mean ± SD)	29.1 ± 6.9		28.2 ± 7.6		0.314
History of miscarriage (*n*, %)	33	30.0	63	24.7	0.292
Maternal diagnosis (*n*, %)					
Gestational hypertensive syndrome	52	47.3	108	42.4	0.385
Urinary tract infection	25	22.7	81	31.8	0.081
Gestational diabetes mellitus	33	30.0	55	21.6	0.084
Gestational age, weeks (mean ± SD)	35.4 ± 2.4		33.1 ± 4.2		< 0.001
Birth weight, grams (mean ± SD)	2545.1 ± 892.5		1988.7 ± 930.8		< 0.001
Female sex (*n*, %)	48	44.0	122	48.6	0.263
5‐min Apgar score (mean ± SD)	8.4 ± 0.9		8.2 ± 1.2		0.061
Neonatal admission diagnosis–ICD‐11 (*n*, %)					
Unspecified respiratory distress of newborn (P22.9)	70	63.6	177	69.4	0.086
Respiratory distress syndrome of newborn (P22.0)	7	6.4	12	4.7	0.643
Other respiratory distress of newborn (P22.8)	4	3.6	13	5.1	0.693
Unspecified cardiovascular disorders originating in the perinatal period (P29.9)	5	4.5	7	2.8	0.376
Length of hospital stay, days (mean ± SD)	7.4 ± 5.7		19.8 ± 15.1		< 0.001
Mortality (*n*, %)	2	1.8	28	12.4	< 0.001

*Note:* Data are presented as mean ± standard deviation (SD) or frequency (*n*, %), as appropriate.

During hospitalization, 255 neonates (69.9%) experienced one or more pDDIs (Table 2). The majority of interactions were classified as risk Category C according to the UpToDate database (88.0% of cases). Based on their mechanisms of action, the identified interactions were grouped by potential clinical outcomes. The most frequent categories included antibiotic‐related pDDIs involving beta‐lactams and aminoglycosides (58.6%); risk of heart rate changes related to the concomitant use of xanthines and vasoactive amines (27.4%); nephrotoxicity associated with furosemide combined with antimicrobials, NSAIDs, or opioids (18.3%); central nervous system (CNS) depression (14.8%); and risk of hypotension (14.8%). Among these, only the interactions potentially related to CNS depression were predominantly classified as risk Category D (12.0%).

**TABLE 2:** Profile of drug interactions classified by severity and potential clinical outcomes based on mechanism of action.

Drug–drug interactions (DDIs)	Neonates		Risk category	Potential clinical outcomes based on mechanism of action					
	*N*	%							
One or more DDIs	255	69.9							
Antibiotic‐related DDIs									
Ampicillin + gentamicin	156	42.7	C	Penicillins may chemically inactivate aminoglycosides and reduce their concentrations under certain conditions. The clinical relevance of this potential interaction depends on the therapeutic and administration context.					
Oxacillin + amikacin	51	14.0	C						
Penicillin + gentamicin	7	1.9	C						
Total	214	58.6							
Heart rate changes‐associated DDIs									
Caffeine + dobutamine	30	8.2	C	Potential cardiotoxicity due to additive effects of sympathomimetic drugs.					
Dobutamine + dopamine	19	5.2	C						
Caffeine + dopamine	18	4.9	C						
Dobutamine + norepinephrine	13	3.6	C						
Caffeine + norepinephrine	10	2.7	C						
Others	10	2.7							
Total	100	27.4							
Nephrotoxicity‐associated DDIs									
Furosemide + fentanyl	24	6.6	C	Loop diuretics may enhance the nephrotoxicity of aminoglycosides and cephalosporins. NSAIDs may reduce their excretion. Thiazide diuretics potentiate NSAID‐induced nephrotoxicity. Opioids may also enhance diuretic nephrotoxicity.					
Amikacin + furosemide	11	3.0	C						
Cefepime + furosemide	11	3.0	C						
Gentamicin + furosemide	10	2.7	C						
Methadone + furosemide	4	1.1	C						
Others	7	1.9							
Total	67	18.3							
Central nervous system depression (CNSD)–associated DDIs									
Fentanyl + phenobarbital	23	6.3	D	Potential additive effect from concurrent use of CNS depressants.					
Fentanyl + fluconazole	19	5.2	D						
Fentanyl + midazolam	8	2.2	D						
Fentanyl + methadone	6	1.6	D						
Morphine + phenobarbital	2	0.5	D						
Others	3	0.9							
Total	61	16.8							
Hypotension‐associated DDIs									
Captopril + phenobarbital	12	3.3	C	Additive hypotensive effects due to concurrent use of antihypertensives.					
Amphotericin B + furosemide	8	2.2	C						
Norepinephrine + furosemide	8	2.2	C						
Hydrochlorothiazide + furosemide	7	1.9	C						
Captopril + furosemide	5	1.4	C						
Others	14	3.8							
Total	54	14.8							
Other potential outcomes									
Amphotericin B + hydrochlorothiazide	4	1.1	C	Metabolic inducers/inhibitors with various clinical consequences.					
Phenytoin + hydrocortisone	3	0.8	C						
Furosemide + dexamethasone	3	0.8	C						
Hydrocortisone + phenobarbital	2	0.5	C						
Captopril + spironolactone	1	0.3	C						
Others	2	0.6							
Total	15	4.2							

*Note:* Distribution of identified drug interactions in neonates, classified according to their clinical relevance (UpToDate classification). Frequencies (*N*) and percentages (%) refer to the total number of neonates exposed to each interaction. Descriptions of potential clinical outcomes were based on information obtained from the UpToDate database.

Incidence rates of DDIs throughout the hospitalization period are shown in Figure 1. Interactions potentially associated with heart rate changes and CNS depression demonstrated incidence rates of 2.67 (95% CI: 2.05–3.29) and 2.60 (95% CI: 1.91–3.28) events per 100 patient‐days, respectively. The median day of detection for these interactions was approximately the 6th day of hospitalization. Both categories showed a decreasing trend over time (*β* = −0.106; *p* < 0.01 and *β* = −0.088; *p* < 0.01, respectively).

**FIGURE 1:**
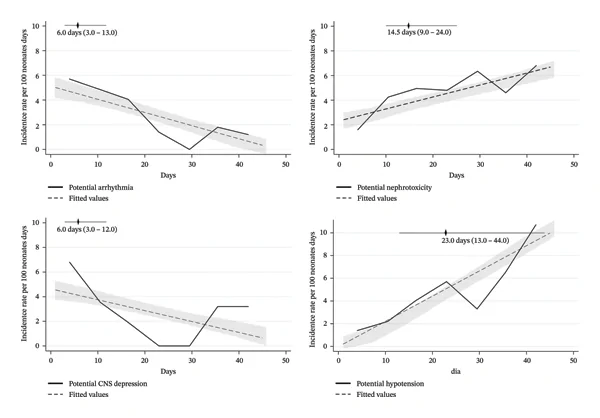
Incidence rate of drug interactions grouped by potential outcomes and median days to detection. Incidence rates of drug interactions associated with heart rate changes, nephrotoxicity, CNS depression, and hypotension throughout hospitalization. Analysis performed using linear mixed‐effects regression: incidence rate as the dependent variable, length of hospitalization and number of medications as fixed‐effect covariates, and intraindividual variability as a random effect. The incidence rate was found to be associated with the length of hospitalization for all categories (*p* < 0.05).

In contrast, DDIs potentially associated with nephrotoxicity and hypotension showed a progressive increase with longer hospitalization: 4.55 (95% CI: 3.91–5.18) and 5.08 (95% CI: 4.05–6.11) events per 100 patient‐days, respectively, with significant upward trends (*β* = 0.097; *p* < 0.01 and *β* = 0.222; *p* < 0.01). The median day of detection for nephrotoxic DDIs was 14 days hospitalization (25th–75th percentile: 9–24), and for those associated with hypotension, 23 days hospitalization (25th–75th percentile: 13–44), indicating that these interactions are more frequently observed in the later stages of hospitalization. Interactions involving aminoglycosides and beta‐lactams were identified and are reported in Table 2 to describe the overall DDI profile; however, they were excluded from the analysis of clinical and laboratory outcomes, since they are associated with therapeutic ineffectiveness rather than measurable clinical or laboratory changes.

Table 3 presents the clinical and laboratory parameters associated with DDIs according to their potential outcomes. DDIs related to heart rate changes were associated with a significant increase in heart rate (158.1 ± 16.7 vs. 148.7 ± 17.0 bpm; *p* < 0.001). Those related to nephrotoxicity were linked to reduced creatinine clearance (14.4 ± 13.5 vs. 25.8 ± 18.2 mL/min/1.73 m^2^; *p* < 0.001), elevated blood urea nitrogen levels (52.9 ± 36.2 vs. 27.6 ± 24.4 mg/dL; *p* < 0.001), and decreased urine output (4.0 ± 2.5 vs. 4.4 ± 2.1 mL/kg/h; *p* = 0.003). Among neonates exposed to pDDIs potentially associated with CNS depression, heart rate was slightly higher (151.9 ± 19.5 vs. 149.0 ± 17.0 bpm; *p* = 0.007), with no significant impact on the respiratory rate. Finally, neonates exposed to interactions potentially associated with hypotension had significantly lower MAP (51.1 ± 11.5 vs. 58.3 ± 11.8 mmHg; *p* < 0.001). In addition, neonates exposed to pDDIs across all evaluated categories had significantly lower gestational age compared to those without such interactions. This difference was observed for DDIs associated with heart rate changes (28.4 ± 4.3 vs. 32.8 ± 4.0 weeks; *p* < 0.001), nephrotoxicity (30.6 ± 5.4 vs. 32.7 ± 4.1 weeks; *p* < 0.001), CNS depression (29.5 ± 5.6 vs. 32.7 ± 4.0 weeks; *p* = 0.007), and hypotension (31.1 ± 5.6 vs. 32.6 ± 4.1 weeks; *p* < 0.001).

**TABLE 3:** Association of drug–drug interactions (DDIs) grouped by potential outcomes on clinical and laboratory parameters.

DDI by potential outcome	No DDI		With DDI		*p* value
	Mean	SD	Mean	SD	
Heart rate changes					
Heart rate (bpm)	148.7	17.0	158.1	16.7	< 0.001
Nephrotoxicity					
Creatinine clearance (mL/min/1.73 m^2^)	25.8	18.2	14.4	13.5	< 0.001
Urine output (mL/kg/hour)	4.4	2.1	4.0	2.5	0.003
Urea (mg/dL)	27.6	24.4	52.9	36.2	< 0.001
CNS depression					
Heart rate (bpm)	149.0	17.0	151.9	19.5	0.007
Respiratory rate (breaths/min)	51.9	8.3	52.5	8.3	0.264
Hypotension					
Mean arterial pressure (mmHg)	58.3	11.8	51.1	11.5	< 0.001

*Note:* Comparative analysis of vital signs and laboratory markers in neonates exposed or not to drug interactions grouped by potential clinical outcomes. Data are presented as mean ± standard deviation (SD). Statistical significance was determined using Student’s *t*‐test.

## 4. Discussion

Based on a prospective cohort of 365 critically ill neonates, we found that more than two‐thirds of patients experienced at least one DDI during hospitalization. When grouped according to their potential outcomes, DDIs associated with heart rate changes and CNS depression was more frequent during the early days of hospitalization. In contrast, DDIs with potential nephrotoxic or hypotensive effects tended to occur more frequently during the later stages of the hospital stay. Notably, neonates exposed to these DDIs exhibited significant alterations in heart rate, blood pressure, and renal function markers.

Identifying DDIs is undoubtedly essential for ensuring the safety of hospitalized patients; however, determining which interactions warrant clinical intervention remains a major challenge in practice. Simply analyzing prescriptions and identifying potentially interacting drug pairs does not necessarily indicate the occurrence of actual harm—such events are thus classified as pDDIs [13]. Furthermore, the DDI profile may vary considerably across clinical settings, depending on patient physiology, clinical diagnoses, and the pharmacological regimens used [14]. This issue is especially complex in critically ill neonates, whose physiological immaturity significantly impacts drug absorption, metabolism, and excretion [15], leading to unpredictable pharmacological responses. The frequent off‐label use of medications and the limited availability of neonatal‐specific data further increase the risk of adverse events and DDIs [3]. As such, identifying DDIs that represent a real risk to neonates remains a complex and ongoing task for NICU teams, particularly given their high incidence.

Polypharmacy is a common feature in NICUs and constitutes a key factor contributing to the high incidence of DDIs in this population [7]. The high frequency and risk profile of DDIs observed in our cohort are consistent with previous studies, in which approximately 66% to 70% of neonates were exposed to at least one DDI [7, 9, 16], with reported rates of major DDIs ranging from 21.3% to 29.1%. However, these studies focused exclusively on *potential* interactions, without assessing associations with clinical or laboratory outcomes. This limitation may contribute to overalerting, potentially undermining clinical decision‐making by intensivists, pharmacists, and nurses.

In our study, DDIs with a potential risk of heart rate changes—particularly those involving xanthines and vasoactive amines, as well as interactions associated with CNS depression (e.g., analgesics and sedatives), tended to occur more frequently at the beginning of treatment, with a subsequent decline in incidence over time. During the early days of hospitalization, prematurity is a common diagnosis, often accompanied by respiratory syndromes and hemodynamic instability, which justifies the widespread use of caffeine and vasoactive drugs [17]. The additive sympathomimetic effect of these DDIs was associated with a significant increase in heart rate. Additionally, during the first weeks, the need for sedation and analgesia for various procedures often leads to the concomitant use of opioids and barbiturates, which may contribute to CNS depression [18]. However, these DDIs were associated with only a small difference in heart rate and no significant difference in respiratory rate.

In contrast, DDIs with potential nephrotoxic and hypotensive effects were more commonly observed after the third week of treatment. Notably, neonates exposed to nephrotoxic DDIs showed a more than one‐third reduction in creatinine clearance and nearly doubled urea levels, indicating significant differences in renal function markers. The primary drug involved in nephrotoxic interactions was furosemide, which is frequently used in conditions such as bronchopulmonary dysplasia, especially in neonates undergoing prolonged mechanical ventilation [19]. A common mechanism underlying these interactions involves furosemide’s inhibitory effect on organic ion transporters, which are responsible for the renal excretion of beta‐lactams and cephalosporins, as well as its ability to promote reabsorption of the cationic forms of aminoglycosides [20]. Given the immaturity of nephrons in preterm infants, such interactions significantly increase the risk of AKI and mortality [21].

Regarding hypotension‐related DDIs, MAP was approximately 12% lower among exposed neonates. However, blood pressure in neonates varies according to gestational and postnatal age [22], which should be considered when interpreting this finding, particularly because these DDIs were more frequently identified later during hospitalization. Hypotension in neonates may compromise blood perfusion to vital organs, particularly the kidneys. A reduction in renal blood flow due to hemodynamic instability can lead to renal ischemia and trigger AKI [23]. Even brief episodes of hypotension may result in inadequate renal perfusion and increase the risk of kidney damage [24].

Although statistically significant, the higher heart rate observed among neonates exposed to these DDIs was not particularly pronounced. However, heart rate variability remains a concern in specific clinical scenarios, such as in neonates with congenital heart disease or sepsis [25], as it may contribute to increased vascular stiffness and impaired cardiac function under stress later in life [26].

By grouping DDIs according to their mechanisms of action and potential outcomes, we were able to temporally situate their occurrence and identify which clinical parameters warrant closer monitoring. Our findings suggest that significant alterations in heart rate during the early days of treatment should raise the suspicion of DDIs as a possible contributing factor, as well as substantial declines in renal function among neonates exposed to furosemide‐related DDIs—particularly in the context of prolonged hospitalization. In contrast, DDIs potentially associated with CNS depression showed limited associations with the clinical parameters evaluated, potentially due to the shorter duration of exposure to the implicated drugs. Nevertheless, these interactions were classified as severe according to the employed database.

This discrepancy between the observed clinical impact and the severity classification of CNS‐related DDIs raises important concerns regarding the reliability of such classifications as tools for clinical decision‐making in NICU settings. Drug interaction databases frequently differ in their categorizations and lack consensus on interaction severity [27], and the criteria used for these classifications are not always supported by robust, population‐specific evidence [25, 28]. The scarcity of studies correlating DDIs with clinical outcomes has led to inappropriate extrapolations based on pharmacokinetic or pharmacodynamic data derived from non‐neonatal populations [29]. Furthermore, some interactions classified as clinically relevant by standard databases may represent established and widely accepted therapeutic strategies in neonatal care, highlighting the need for cautious interpretation of DDI alerts and severity ratings within the clinical context.

This prospective study enabled continuous monitoring of a large number of neonates throughout hospitalization and is the first to correlate groups of DDIs with clinical and laboratory outcomes in preterm infants in the NICU. The findings may support healthcare teams in prioritizing the monitoring of critical parameters and guiding therapeutic decisions based on the clinical relevance of specific DDIs.

This study has several important limitations. As it was conducted in a single institution, the findings may not be generalizable to other NICUs with different care profiles. Additionally, it was not possible to definitively determine whether the observed clinical effects were attributable to individual drugs or to DDIs, given the therapeutic complexity of the patients. The use of a single drug interaction database, primarily based on adult evidence, may have limited the accuracy of DDI classification in the neonatal population. Furthermore, the lack of adjustment for potential confounders restricts the ability to infer causality. This limitation is partly related to the exploratory nature of the study, which aimed to identify potential associations between groups of DDIs and measurable clinical parameters, rather than to establish causal relationships. Renal function was evaluated using estimated creatinine clearance without accounting for the expected postnatal maturation of glomerular filtration. Because renal function changes rapidly after birth and is influenced by both gestational and postnatal age, differences in creatinine clearance may partly reflect physiological renal maturation rather than DDI exposure. Therefore, these findings should be interpreted as exploratory associations and not as evidence of AKI. Given the heterogeneity of the identified DDIs and their distinct mechanisms, more robust evaluation of their clinical impact would require outcome‐specific approaches with more detailed and targeted data collection. In addition, given the exploratory nature of the study, more specific clinical outcomes such as the occurrence of arrhythmias or seizures were not systematically evaluated. Future studies adopting more comprehensive and tailored methodological approaches are warranted to better characterize the clinical relevance and impact of DDIs in this population.

Nonetheless, this is the first prospective study to perform daily pharmacotherapy monitoring and to group DDIs according to clinical and laboratory outcomes in preterm neonates. The results may assist clinical teams in identifying interactions that merit closer attention and tailoring interventions based on actual clinical relevance. Future multicenter studies using adjusted multivariate analyses and causal inference frameworks are warranted to strengthen the evidence on the true clinical impact of DDIs in neonates.

This prospective study identified a high frequency of DDIs among neonates admitted to a NICU. When classified according to their mechanisms of action and clinical‐laboratory outcomes, DDIs were grouped by their potential to cause heart rate changes, nephrotoxicity, CNS depression, and hypotension. DDIs related to heart rate changes and CNS depression were more common during the early days of hospitalization, whereas nephrotoxic and hypotensive interactions tended to occur in later stages. DDIs associated with heart rate changes, nephrotoxicity, and hypotension were associated with measurable differences in heart rate, renal function markers, and MAP, respectively.

## 5. Study Highlights

Neonates admitted to NICUs are commonly exposed to multiple medications, increasing the risk of pDDIs. Despite the high frequency of such interactions, evidence describing their clinical consequences in this vulnerable population remains limited. This study investigated the occurrence of pDDIs in 365 critically ill neonates and assessed their potential clinical impact. During hospitalization, 69.9% of the patients experienced at least one pDDI, with a predominance of interactions classified as moderate risk (Category C). The most frequent interactions involved potential risks of heart rate changes, nephrotoxicity, CNS depression, and hypotension and were associated with alterations in clinical parameters. These findings highlight the importance of systematic DDI monitoring in neonatology and emphasize their potential clinical relevance to patients’ clinical stability. Implementing DDI surveillance and management strategies may contribute to safer and more personalized pharmacotherapy in NICUs, while also providing valuable insights for clinical pharmacology and translational science in neonatal care.

## Funding

This study was funded by Coordenação de Aperfeiçoamento de Pessoal de Nível Superior (CAPES) (Finance Code 001).

## Disclosure

All authors have read and approved the final version of the manuscript; the corresponding author had full access to all of the data in this study and takes complete responsibility for the integrity of the data and the accuracy of the data analysis. The funding source had no role in the study design, data collection, analysis or interpretation of data, writing of the manuscript, or the decision to submit the article for publication.

Rand Randall Martins affirms that this manuscript is an honest, accurate, and transparent account of the study being reported; that no important aspects of the study have been omitted; and that any discrepancies from the study as planned have been explained.

## Ethics Statement

It was approved by the Ethics Committee of Maternidade Escola Januário Cicco (approval no. 5867920), in accordance with Resolution 466/12. Written consent was obtained from legal guardians.

## Conflicts of Interest

The authors declare no conflicts of interest.

## Data Availability

The data that support the findings of this study are not publicly available due to ethical and privacy restrictions related to patient confidentiality. Deidentified data may be made available by the corresponding author upon reasonable request and subject to approval by the relevant ethics committee and institutional regulations.
